# Evaluation of curcumin and copper acetate against *Salmonella* Typhimurium infection, intestinal permeability, and cecal microbiota composition in broiler chickens

**DOI:** 10.1186/s40104-021-00545-7

**Published:** 2021-02-05

**Authors:** Anaisa A. Leyva-Diaz, Daniel Hernandez-Patlan, Bruno Solis-Cruz, Bishnu Adhikari, Young Min Kwon, Juan D. Latorre, Xochitl Hernandez-Velasco, Benjamin Fuente-Martinez, Billy M. Hargis, Raquel Lopez-Arellano, Guillermo Tellez-Isaias

**Affiliations:** 1 Departamento de Medicina y Zootecnia de Aves, Facultad de Medicina Veterinaria y Zootecnia, UNAM, 04510 Ciudad de Mexico, Mexico; 2 Laboratorio 5: LEDEFAR, Unidad de Investigacion Multidisciplinaria, Facultad de Estudios Superiores (FES) Cuautitlan, Universidad Nacional Autonoma de Mexico (UNAM), 54714 Cuautitlan Izcalli, Mexico; 3 Department of Poultry Science, Center of Excellence for Poultry Science, University of Arkansas, 1260 W. Maple, POSC 0-114, Fayetteville, AR 72704 USA; 4 Centro de Ensenanza, Investigacion y Extension en Produccion Avicola, Facultad de Medicina Veterinaria y Zootecnia, UNAM, Ciudad de Mexico, Mexico

**Keywords:** Broiler chickens, Copper acetate, Curcumin, Intestinal permeability, Microbiota composition, *Salmonella* Typhimurium

## Abstract

**Background:**

Interest in the use of natural feed additives as an alternative to antimicrobials in the poultry industry has increased in recent years because of the risk of bacterial resistance. One of the most studied groups are polyphenolic compounds, given their advantages over other types of additives and their easy potentiation of effects when complexes are formed with metal ions. Therefore, the objective of the present study was to evaluate the impact of dietary supplementation of copper acetate (CA), curcumin (CR), and their combination (CA-CR) against *Salmonella* Typhimurium colonization, intestinal permeability, and cecal microbiota composition in broiler chickens through a laboratory *Salmonella* infection model. *S.* Typhimurium recovery was determined on day 10 post-challenge by isolating *Salmonella* in homogenates of the right cecal tonsil (12 chickens per group) on Xylose Lysine Tergitol-4 (XLT-4) with novobiocin and nalidixic acid. Intestinal integrity was indirectly determined by the fluorometric measurement of fluorescein isothiocyanate dextran (FITC-d) in serum samples from blood obtained on d 10 post-*S.* Typhimurium challenge. Finally, microbiota analysis was performed using the content of the left caecal tonsil of 5 chickens per group by sequencing V4 region of 16S rRNA gene.

**Results:**

The results showed that in two independent studies, all experimental treatments were able to significantly reduce the *S*. Typhimurium colonization in cecal tonsils (CT, *P* < 0.0001) compared to the positive control (PC) group. However, only CA-CR was the most effective treatment in reducing *S*. Typhimurium counts in both independent studies. Furthermore, the serum fluorescein isothiocyanate dextran (FITC-d) concentration in chickens treated with CR was significantly lower when compared to PC (*P* = 0.0084), which is related to a decrease in intestinal permeability and therefore intestinal integrity. The effect of dietary treatments in reducing *Salmonella* was further supported by the analysis of 16S rRNA gene sequences using Linear discriminant analysis effect size (LEfSe) since *Salmonella* was significantly enriched in PC group (LDA score > 2.0 and *P* < 0.05) compared to other groups. In addition, *Coprobacillus*, *Eubacterium*, and *Clostridium* were significantly higher in the PC group compared to other treatment groups. On the contrary, *Fecalibacterium* and *Enterococcus* in CR, unknown genus of Erysipelotrichaceae at CA-CR, and unknown genus of Lachnospiraceae at CA were significantly more abundant respectively.

**Conclusions:**

CR treatment was the most effective treatment to reduce *S*. Typhimurium intestinal colonization and maintain better intestinal homeostasis which might be achieved through modulation of cecal microbiota.

**Supplementary Information:**

The online version contains supplementary material available at 10.1186/s40104-021-00545-7.

## Background

*Salmonella*, a Gram-negative intracellular bacteria, is a food-borne pathogen that can cause gastroenteritis and severe systemic infections in humans [1–3], as well as significant economic losses in poultry production because it can cause high mortality and affect growth performance parameters in broiler chickens [4, 5]. Recently, it has been reported that the global incidence of salmonellosis cases has increased, estimating that of the approximately 94 million reported cases, 155,000 lead to death each year [6]. Furthermore, the estimated costs of medical expenses, sick leaves, and loss of productivity range from US$1.3 to US$4.0 billion a year in the United States of America (USA) [7].

It is known that young chickens are more susceptible to colonization by *Salmonella* [8], with the cecum being the target site for establishing chronic infection [9]. Although the genus *Salmonella* consists of more than 2600 serovars, the most common serotypes isolated from chicken-associated outbreaks are *Salmonella enterica* serovar Enteritidis (20%) and *Salmonella enterica* serovar Typhimurium (17%) [10, 11].

Nowadays, due to regulations on the use of antibiotics in poultry production derived from the problems of bacterial resistance, and considering that *S*. Typhimurium is a bacterium capable of developing antimicrobial resistance more quickly than other *Salmonella* species [12, 13], several strategies have been proposed to treat and control *Salmonella* infections [14]. Among the large number of alternatives that have been tested in recent years, it has been reported that the combination of polyphenolic compounds with metal ions, such as copper, have potentiated antioxidant, anti-inflammatory and antimicrobial effects, having the additional advantage of reducing toxicity of metal ions due to complex formation [15, 16].

Copper compounds such as copper acetate (CA) are believed to promote growth by regulating gastrointestinal microbiota through bactericidal and bacteriostatic effects [17]. The mechanisms that explain the antimicrobial effect of the copper ion are related to direct damage to the bacteria’s membrane, which generates a loss of membrane potential and cytoplasmic content. Furthermore, reactive oxygen species produced by copper ions induce further damage to cellular structures and even DNA degradation [18].

Another alternative is curcumin (CR), a mixture of polyphenolic compounds obtained from the rhizome or root of the *Curcuma longa* plant, member of the Zingiberaceae or ginger family that is characterized by its excellent antioxidant, anti-inflammatory, and immunomodulatory properties, as well as its antimicrobial and growth-promoting effects [19–21]. However, an essential limitation of CR is its low solubility and permeability. Recent studies performed by our laboratories have shown that the use of solid dispersions with polyvinylpyrrolidone can increase these biopharmaceutical properties [22, 23]. Therefore, the objective of the present study was to evaluate the effect of dietary supplementation of CA, CR, and their combination (CA-CR) against *S*. Typhimurium colonization, intestinal permeability, and cecal microbiota composition using a model of *S.* Typhimurium infection in broiler chickens.

## Methods

### Preparation of experimental treatments and diets

CR treatment consisted of a solid dispersion of curcumin with polyvinylpyrrolidone in a 1:9 ratio previously described [22, 23], CA treatment was copper(II) acetate hydrate (98%, Catalog No. 341746*,* Sigma), and CA-CR treatment consisted of a mixture of the previous treatments. Solid dispersion of curcumin was prepared by dissolving 1 part of curcumin in 9 parts of a polyvinylpyrrolidone (PVP) K30 solution, followed by water evaporation at 40 °C and sieving. Mash corn-soybean-based broiler starter basal diet was formulated to approximate the nutritional requirements of broiler chickens, as recommended by the National Research Council [24] and then adjusted to breeder’s recommendations [25]. No antibiotics, coccidiostats or enzymes were added to the feed (Table 1). All animal handling procedures complied with the Institutional Animal Care and Use Committee (IACUC) at the University of Arkansas, Fayetteville (protocol #18029).

**Table 1:** Ingredient composition and nutrient content of a basal starter diet used in the experiment on as-fed basis

Item	Corn soybean-based diet
Ingredients, %	
Corn	57.34
Soybean meal	34.66
Poultry fat	3.45
Dicalcium phosphate	1.86
Calcium carbonate	0.99
Salt	0.38
*DL*-Methionine	0.33
*L*-Lysine HCl	0.31
Threonine	0.16
Vitamin premix^a^	0.20
Mineral premix^b^	0.10
Choline chloride 60%	0.20
Calculated analysis	
Metabolizable energy, kcal/kg	3035
Crude protein, %	22.16
Ether extract, %	5.68
Lysine, %	1.35
Methionine, %	0.64
Methionine + cystine, %	0.99
Threonine, %	0.92
Tryptophan, %	0.28
Total calcium, %	0.90
Available phosphorus, %	0.45
Determined analysis	
Crude protein, %	21.15
Ether extract, %	6.05
Calcium, %	0.94
Phosphorus, %	0.73

^a^Vitamin premix supplied per kg of diet: retinol, 6 mg; cholecalciferol, 150 μg; *DL*-α-tocopherol, 67.5 mg; menadione, 9 mg; thiamine, 3 mg; riboflavin, 12 mg; pantothenic acid, 18 mg; niacin, 60 mg; pyridoxine, 5 mg; folic acid, 2 mg; biotin, 0.3 mg; cyanocobalamin, 0.4 mg

^b^Mineral premix supplied per kg of diet: Mn, 120 mg; Zn, 100 mg; Fe, 120 mg; copper, 10 to 15 mg; iodine, 0.7 mg; selenium, 0.2 mg; and cobalt, 0.2 mg

### *Salmonella* strain and culture conditions

The poultry strain of *Salmonella* Typhimurium (PHL-2020) was obtained from the USDA National Veterinary Services Laboratory (Ames, IA, USA). This strain was selected for resistance to 25 μg/mL of novobiocin (NO, Catalog No. N-1628, Sigma) and 20 μg/mL of nalidixic acid (NA, Catalog No. N-4382, Sigma) in our laboratory. In the present study, 100 μL of *S.* Typhimurium from a frozen aliquot was added to 10 mL of tryptic soy broth (TSB, Catalog No. 22092, Sigma, St. Louis, MO, USA), incubated at 37 °C for 8 h, and passed three times every 8 h to ensure that all bacteria were in log phase as previously described [26]. Post-incubation, bacteria were washed three times with sterile 0.9% saline by centrifugation at 1864×*g* for 10 min, reconstituted in saline, quantified by densitometry with a spectrophotometer (Spectronic 20 DC, Spectronic Instruments Thermo Scientific, Rochester, NY, USA) and finally diluted to an approximate concentration of 10^4^ CFU/mL. Levels of *S.* Typhimurium were further verified by serial dilutions and plated on brilliant green agar (BGA, Catalog No. 70134, Sigma, St. Louis, MO, USA) with NO and NA for enumeration of actual CFU used in the experiment.

### Animal source and experimental design

In the present study, two independent trials with 75 day-of-hatch male Cobb-Vantress broiler chickens (Fayetteville, AR, USA) were conducted. Chicks were individually weighed and randomly assigned to one of five groups (*n* = 15 chickens/group): 1) Negative control (NC, basal diet); 2) Positive control (PC, basal diet + challenged with 10^4^ CFU of *S.* Typhimurium per bird on hatching day); 3) CA (basal diet supplemented with 250 mg/kg of copper(II) acetate hydrate + challenged with 10^4^ CFU of *S.* Typhimurium per bird on hatching day); 4) CR (basal diet supplemented with 0.2% curcumin + challenged with 10^4^ CFU of *S.* Typhimurium per bird on hatching day); and 5) CA-CR (basal diet supplemented with 250 mg/kg of copper (II) acetate hydrate and 0.2% curcumin + challenged with 10^4^ CFU of *S.* Typhimurium per bird on hatching day). In both trials, chicks were raised in floor pens (118 in × 59 in), provided with their diet, water ad libitum, and maintained at an age-appropriate temperature during all experiments. Body weight (BW) and body weight gain (BWG) were evaluated at 10 days of age. On day ten post-*S.* Typhimurium challenge, chickens were given an appropriate dose of fluorescein isothiocyanate dextran (FITC-d) by oral gavage 1 h before the chickens were euthanized by CO_2_ inhalation and only the right cecal tonsil (CT) from 12 broilers per group were aseptically collected for *S.* Typhimurium recovery. Furthermore, blood samples were also collected from the femoral vein for the determination of FITC-d. The concentration of FITC-d administered was calculated based on group body weight at day nine post-*S.* Typhimurium challenge. For microbiota analysis, the content of the left CT was collected aseptically and stored at − 20 °C until analysis. The number of broilers chosen per group for each determination was based on reproducible results from experiments previously described and published by our laboratory [22, 23].

### *Salmonella* recovery

In both independent trials, the right CT samples from 12 chickens per group were individually homogenized and diluted with saline (1:4; w/v), and 10-fold dilutions were plated on Xylose Lysine Tergitol-4 (XLT-4, Catalog No. 223410, BD DifcoTM) with NO and NA for *S.* Typhimurium recovery. Plates were incubated at 37 °C for 24 h to enumerate total *S.* Typhimurium colony-forming units. Subsequently, the CT samples were enriched in 2× concentrated tetrathionate enrichment broth and further incubated at 37 °C for 24 h. Enrichment samples were streaked onto XLT-4 with NO and NA selective media for confirmation of *Salmonella* presence. Samples that were negative in the plate dilution method but positive after enrichment with tetrathionate received an arbitrary value of 500 CFU/g (lower limit of detection).

### Serum determination of FITC-d leakage

FITC-d (MW 3–5 kDa; Sigma-Aldrich Co., St. Louis, MO, USA) was provided by oral gavage to 12 broiler chickens from each group at a dose of 8.32 mg/kg of body weight 1 h before the chicks were euthanized by CO_2_ inhalation with the purpose of evaluating the paracellular transport and mucosal barrier dysfunction [27, 28]. Three remaining broiler chickens of each group were used as controls. The blood samples were centrifuged (1000×*g* for 15 min) to separate the serum. Then, serum samples obtained were diluted (1:5) and measured fluorometrically at an excitation wavelength of 485 nm and an emission wavelength of 528 nm (Synergy HT, Multi-mode microplate reader, BioTek Instruments, Inc., VT, USA) to determine the serum FITC-d levels [29].

### Microbiota analysis

#### DNA extraction, PCR, and library preparation for sequencing

V4 region of 16S rRNA gene from the genomic DNA of each of the 25 samples of cecal content (5 samples per group × 5 groups) was amplified using the primers 515F [30] and 806R [31]. The library of amplicons for DNA sequencing was prepared according to the 16S Illumina PCR protocol described in the Earth Microbiome Project (http://www.earthmicrobiome.org) with slight modifications [32]. In brief, Q5® High-Fidelity DNA Polymerase user guide protocol (New England Biolabs, Catalog No. M0491S) was used to conduct PCR in a 25-μL final reaction volume via 30 amplification cycles. The length of the amplified product was confirmed with 1% agarose gel electrophoresis, and equal amount (~ 300 ng) of the amplicons from each sample as measured by Qubit dsDNA BR Assay Kit (ThermoFisher Scientific, Catalog No. Q32850) were pooled together. The pooled amplicons were finally run on 1% agarose gel electrophoresis, purified using Zymoclean Gel DNA Recovery Kit (Zymo Research, Catalog No. D4007), and sequenced with Illumina MiSeq paired-end 300 cycle options at Admera Health, LLC (New Jersey, USA). Despite the small number of samples analyzed, previous studies using 6 samples have shown reliable results since even omitting outliers the trend is the same [33, 34].

#### Amplicons sequence analysis

Nebula cloud computing platform of the University of Arkansas was used to process raw sequencing reads in QIIME 2 version 2018.8 utilizing the pipelines developed for paired-end data types [35]. In sum, “demux emp-paired” method of q2-demux plugin was used to demultiplex sequencing reads, followed by quality filtering and denoising with “dada2 denoise-paired” method of q2-dada2 [36] plugin available at QIIME 2. The truncation length of forward and reverse reads were set at 220 and 200 bp, respectively, which was based on the quality score criteria (≥30). Taxonomic assignment was performed using a Naive Bayes classifier pre-trained with Greengenes (version 13.8) 99% OTUs [37] and q2-feature-classifier plugin, where the sequences have been trimmed to include only the V4 region of the 16S rRNA gene region which is defined by the 515F/806R primer pair. We detected the sequence reads assigned to Chloroplast and Mitochondria, which were subsequencingtly removed using taxonomy-based filtering option in QIIME2. The core-metrics-phylogenetic method at a sampling depth of 69,566 was used to analyze Alpha and Beta diversity. Observed OTUs were used to calculate alpha diversity, while weighted UniFrac distance and unweighted UniFrac distance metrics were used for beta diversity analysis. All figures were created using ggplot2 packages of R [38].

### Data and statistical analysis

After demonstrating that data from *S.* Typhimurium counts (log_10_ CFU/g), serum determination of FITC-d leakage, BW and BWG presented a normal distribution and homogeneity of the variances using the Levene and Ryan-Joiner procedures, respectively, these data were subjected to analysis of variance (ANOVA) as a completely randomized design using the General Linear Models procedure of Statistical Analysis System (SAS®) [39]. Significant differences among the means were determined by Duncan’s multiple range test at *p* < 0.05. Enrichment data were expressed as positive/total chickens (%), and the percentage of *S.* Typhimurium positive samples were compared by a Chi-square test of independence [40], testing all possible combinations to determine the significance (*P* < 0.05).

Statistical differences of bacterial taxa at different levels (family and genus) among treatment groups were determined using linear discriminant analysis effect size (LEfSe) using all against all comparison mode, where the level of significance was set at LDA score > 2.0 and *P* < 0.05 [41]. The significant differences in alpha diversity were calculated using an alpha-group-significance command of QIIME2, which is based on the Kruskal-Wallis test. Statistical differences in beta diversity among groups were calculated by PERMANOVA [41] test using a beta-group-significance command of QIIME2 with a pairwise option. For both diversities analysis, the corrected *P* values for multiple comparisons (q) were used to report a significant difference between the two groups, where the level of significance was set at q < 0.05.

## Results

The results of the antimicrobial effect of CA, CR, and CA-CR on *S*. Typhimurium colonization in the CT of broiler chickens in trial one and trial two are summarized in Table 2. In both trials, all experimental treatments were able to significantly reduce the *S*. Typhimurium colonization in CT (*P* < 0.0001) when compared to the PC group. However, CR and CA-CR were the most effective treatments, since they reduced the colonization of *S*. Typhimurium more than 2.1 and 2.3 log_10_ (*P* = 0.002 and *P* = 0.008), respectively, compared to PC. Although the data are not presented in Table 2, the presence of *Salmonella* was confirmed in all the samples of the experimental groups, with the exception of NC.

**Table 2:** Evaluation of copper acetate (CA), curcumin (CR) and copper acetate – curcumin (CA-CR) on cecal tonsils (CT) colonization of *Salmonella* Typhimurium^1^ and serum concentration of fluorescein isothiocyanate-dextran (FITC-d) in broiler chickens on day ten post-*S*. Typhimurium challenge^2^ in trial 1 and trial 2

Treatments	CT, log_**10**_ CFU/g	FITC-d, ng/mL
	Trial 1	
CTRL (−)	0.00 ± 0.00 ^d^	17.03 ± 5.44 ^b^
CTRL (+)	6.18 ± 0.33 ^a^	54.99 ± 10.51 ^a^
CA	4.99 ± 0.32 ^b^	35.19 ± 8.80 ^ab^
CR	3.92 ± 0.55 ^bc^	17.60 ± 7.50 ^b^
CA-CR	3.76 ± 0.54 ^c^	32.99 ± 10.34 ^ab^
SEM^3^	0.33	4.18
*P*-value	0.000	0.020
	Trial 2	
CTRL (−)	0.00 ± 0.00 ^d^	19.80 ± 9.26 ^b^
CTRL (+)	6.09 ± 0.276 ^a^	59.38 ± 9.81 ^a^
CA	4.94 ± 0.32 ^b^	32.99 ± 11.31 ^ab^
CR	3.91 ± 0.19 ^c^	15.40 ± 7.60 ^b^
CA-CR	3.78 ± 0.31 ^c^	39.59 ± 15.06 ^ab^
SEM^3^	0.30	5.13
*P*-value	0.000	0.048

^1^ Data expressed in log_10_ CFU/g of tissue. Mean ± SE from 12 chickens. ^a–d ^Values within treatments columns for each treatment with different superscripts differ significantly (*P* < 0 .05)

^2^ Chickens were orally gavaged with 10^4^ CFU of *S*. Typhimurium per chicken at 1 day old, samples were collected at day 10 post-challenge

^3^ Standard error of the means

Table 2 shows the results of the dietary administration of CA, CR, and CA-CR on serum FITC-d concentration in broiler chickens on day ten post-*S*. Typhimurium challenge. In both trials, there were no significant differences in the serum FITC-d concentration when the CA and CA-CR groups were compared to groups PC and NC. However, the serum FITC-d concentration in chickens treated with CR was significantly lower when compared to PC (*P* = 0.008), but there were no significant differences when compared to NC.

The effect of the dietary inclusion of the treatment into the feed on the body weight at day 10, as well as the body weight gained of the broilers in each of the independent studies is summarized in Table 3. At the beginning of the experiment, no significant differences were shown in the weights of the broilers. However, at day 10, only the group treated with CR presented significant differences in BW compared to PC. Furthermore, at the end of the experiments, BWG increased significantly in the group treated with CR when compared to PC. Although the groups treated with CA and CA-CR did not show significant differences in BW on day 10 and BWG, a tendency to improve these parameters was observed in comparison with PC (*P* = 0.085 and *P* = 0.119, respectively).

**Table 3:** Evaluation of copper acetate (CA), curcumin (CR) and copper acetate-curcumin (CA-CR) on body weight (BW), body weight gain (BWG), feed intake (FI) and feed conversion ratio (FCR) in broiler chickens on day ten post-*S.* Typhimurium challenge in trial 1 and trial 2^1^

Treatments	BW, g/broiler (D 0)	BW, g/broiler (D 10)	BWG, g/broiler (D 0–10)
	Trial 1		
CTRL (−)	40.60 ± 0.57	232.33 ± 9.46 ^ab^	191.73 ± 9.39 ^ab^
CTRL (+)	40.13 ± 0.86	204.97 ± 10.06 ^b^	164.83 ± 10.05 ^b^
CA	40.67 ± 0.70	227.50 ± 9.32 ^ab^	186.83 ± 9.40 ^ab^
CR	40.87 ± 1.01	237.93 ± 7.80 ^a^	197.07 ± 7.60 ^a^
CA-CR	40.47 ± 0.62	226.8 ± 8.75 ^ab^	186.33 ± 8.83 ^ab^
SEM^2^	0.34	4.17	4.15
*P*-value	0.973	0.122	0.134
	Trial 2		
CTRL (−)	40.93 ± 0.64	231.83 ± 7.54 ^ab^	190.90 ± 7.28 ^ab^
CTRL (+)	40.07 ± 0.95	205.6 ± 7.75 ^b^	165.53 ± 7.90 ^b^
CA	40.40 ± 0.79	226.83 ± 8.82 ^ab^	186.43 ± 8.66 ^ab^
CR	41.27 ± 0.71	236.63 ± 8.00 ^a^	195.37 ± 8.04 ^a^
CA-CR	40.40 ± 0.84	226.6 ± 10.51 ^ab^	186.2 ± 10.68 ^ab^
SEM^2^	0.35	3.94	3.92
*P*-value	0.833	0.119	0.143

^1^ Data expressed as mean ± SE from 15 chickens. ^a -b^ Values within columns with different superscripts differ significantly (*P* < 0.05)

^2^ Standard error of the means

**Fig. 1:**
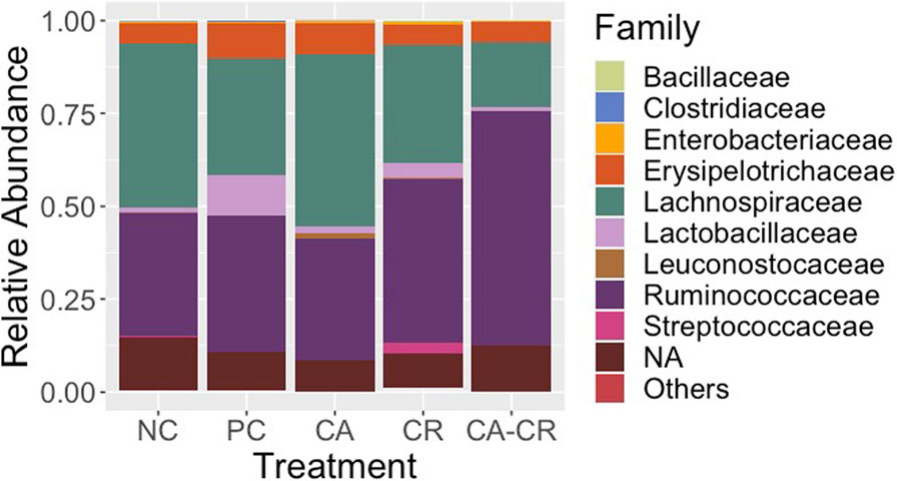
Taxonomic composition of the cecal microbiota in the different treatments at family level. NC: negative control, PC: positive control, CA: cooper acetate, CR: curcumin, and CA-CR: copper acetate-curcumin. “NA” refers to the bacterial taxa that were not assigned to the family level but were assigned at the higher taxonomic level. “Others” represent the minor bacterial families whose relative abundance were < 0.1%

**Fig. 2:**
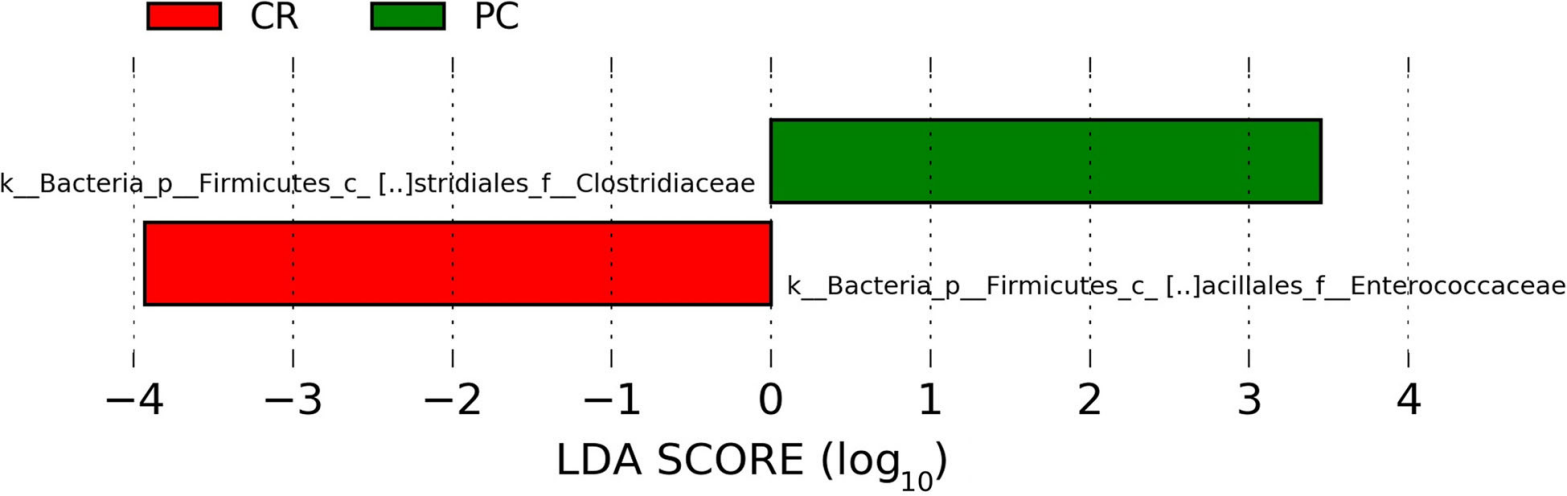
Taxonomic difference of the main families in the microbiota between the curcumin-treated group (CR) and the positive control (PC)

**Fig. 3:**
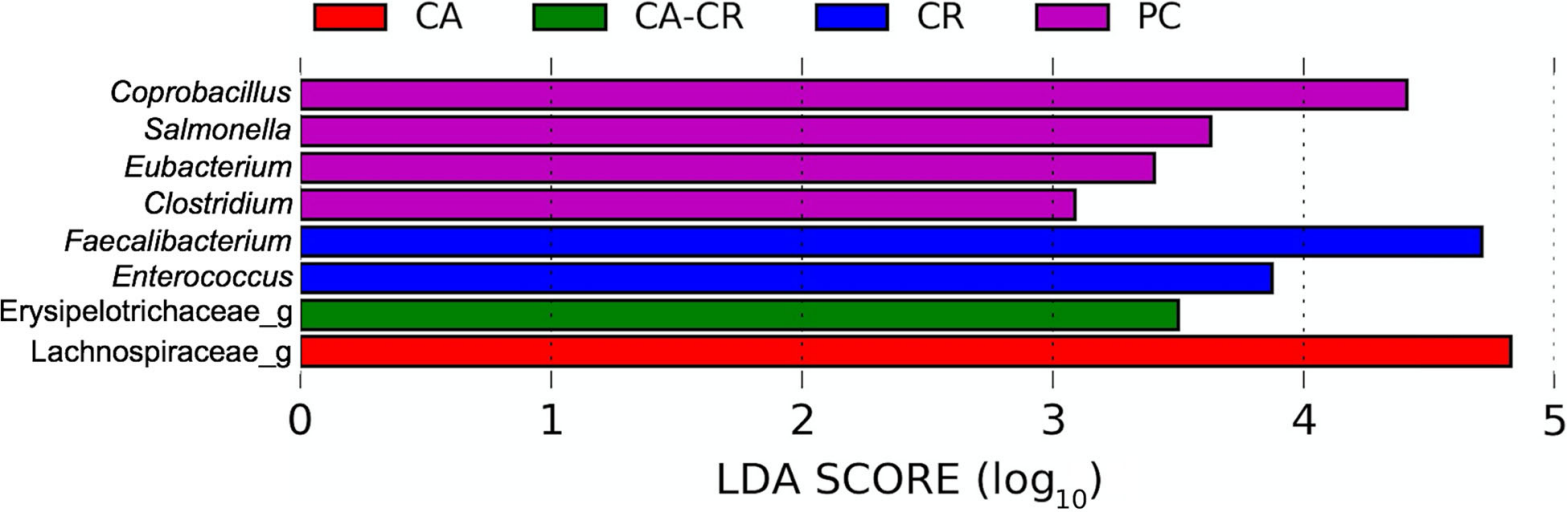
Taxonomic composition of the microbiota in the groups treated at the genus level. PC: positive control, CA: cooper acetate, CR: curcumin, and CA-CR: copper acetate – curcumin

Cecal microbiota was analyzed in samples collected from day ten post-*S*. Typhimurium challenged birds. The relative abundance of different bacterial families recovered across different groups is shown in Fig. 1. In all five groups, either Ruminococcaceae or Lachnospiraceae were the most predominant families. Lachnospiraceae was the most dominant bacterial family in NC (44.10%) and CA (46.37%) followed by Ruminococcaceae (NC, 33.23%; CA, 32.86%). However, Ruminococcaceae was found the highest in PC (36.50%), CR (44.17%), and CA-CR (63.12%) followed by Lachnospiraceae (PC, 31.37%; CR, 31.78%; CA-CR, 17.69%). LEfSe analysis (LDA score > 2.0 and *P* < 0.05) revealed some important differentially abundant taxa at both bacterial family and genus level. As shown in Fig. 2, Enterococcaceae was significantly higher in the PC group while Clostridiaceae was significantly enriched in CR group. Furthermore, at genus level, *Salmonella, Coprobacillus, Eubacterium,* and *Clostridium* were significantly abundant in PC, while the genera *Fecalibacterium* and *Enterococcus* were significantly enriched in CR group and the unknown genera that belong to Erysipelotrichaceae and Lachnospiraceae were significantly enriched in CA-CR and CA groups, respectively (Fig. 3). To assess the accuracy of the taxonomic assignment of the reads matching to genus Salmonella, we did BLAST analysis using the corresponding amplicon sequence variant (ASV) sequence as shown in Supplementary Materials, which strongly support correct identification of genus *Salmonella*.

**Fig. 4:**
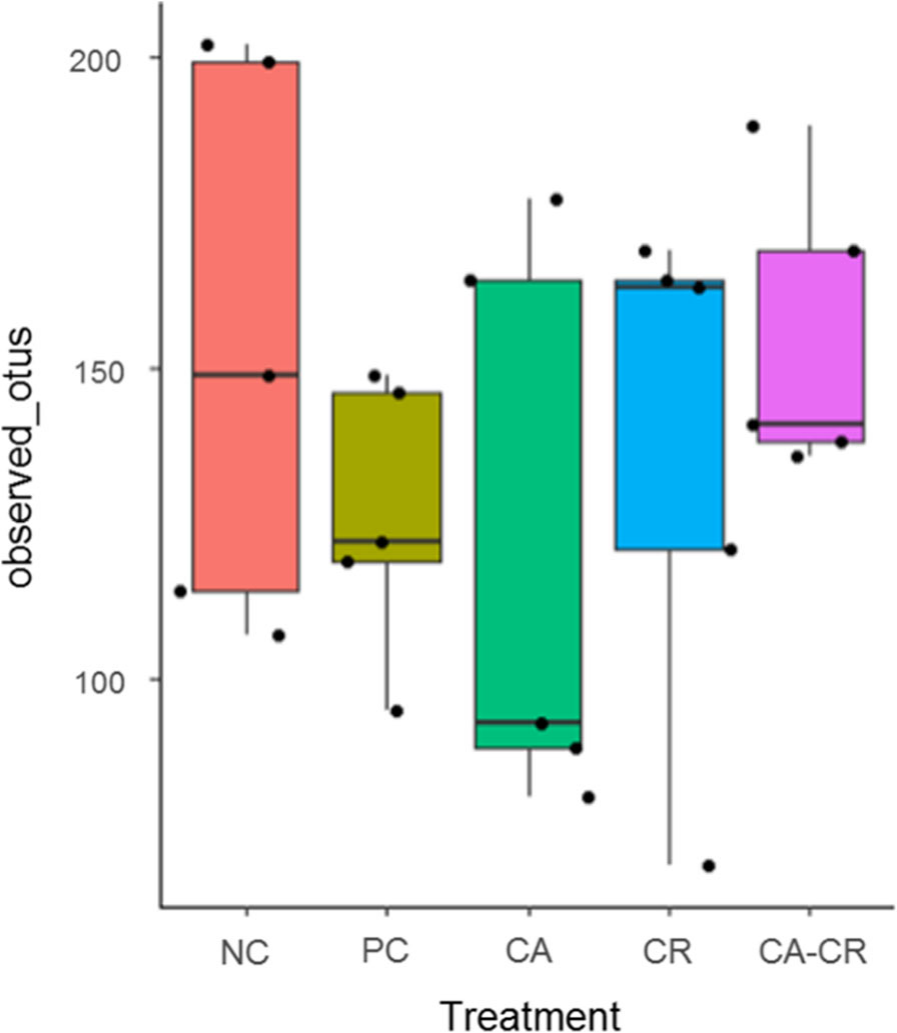
Comparison of the alpha diversities among the groups as measured by observed OTUs

Alpha diversity analysis among the groups, as measured by observed OTUs is shown in Fig. 4. Although there were no significant differences among the groups (Kruskal-Wallis test; *P* > 0.05), it was observed that PC group presented a numerically lower diversity compared to the other groups.

**Fig. 5:**
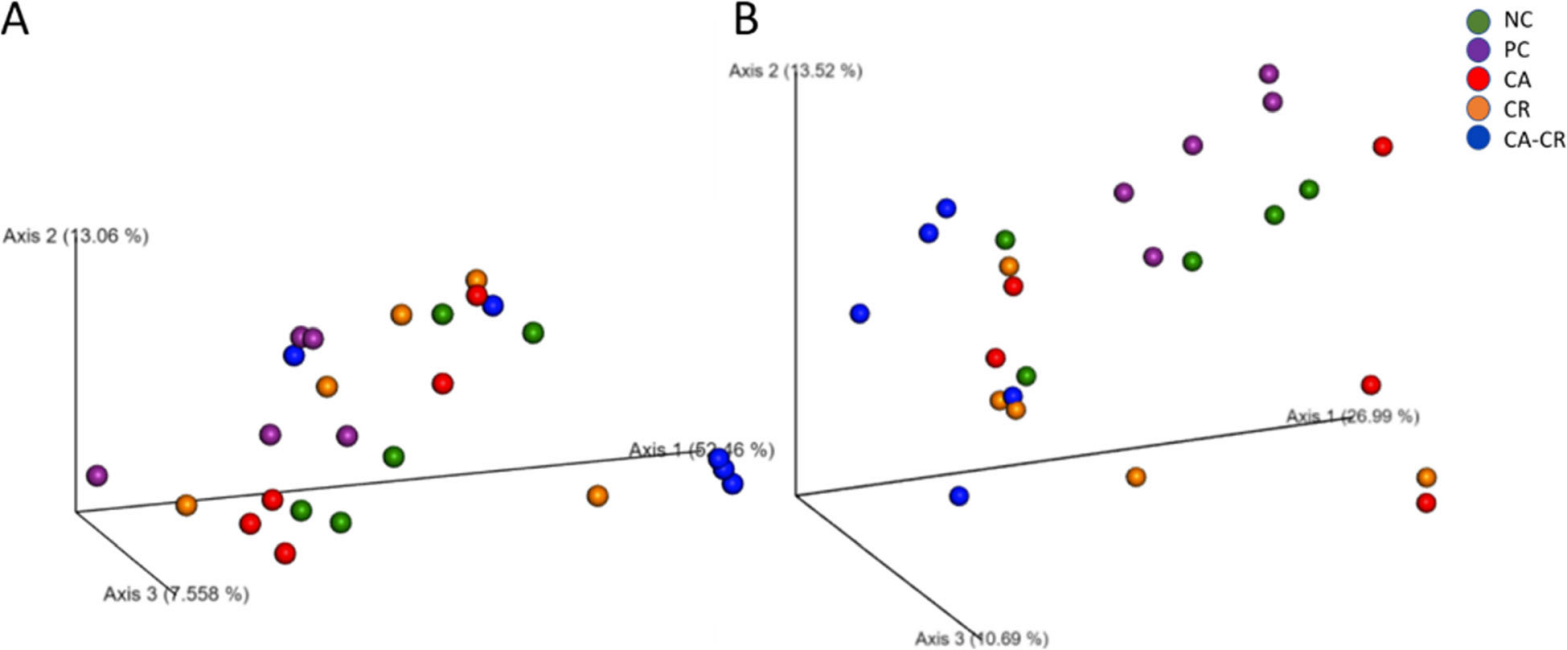
PCoA plots showing beta diversity among the groups according to **a** weighted UniFrac distance metric and **b** unweighted UniFrac distance metric

Beta diversities among different groups as measured by weighted and unweighted UniFrac distance metrics are illustrated in PCoA plots (Fig. 5a and b, respectively). Results of the Permutational multivariate analysis of variance (PERMANOVA) showed that there were no significant differences in the structure of the microbial community among the groups at q < 0.05.

## Discussion

Modern animal production has been changing in recent years due to the problems of bacterial resistance derived from the overuse of antimicrobials for prophylactic and growth promotion purposes [42, 43]. In this regard, many investigations have focused on probiotics, prebiotics, enzymes, acidifiers, plant extracts, and some metals (copper and zinc) as feed additives, given their antimicrobial properties and effects in promoting growth, mainly [44]. In the present study, selection of CA was based on its advantages over inorganic sources since it has been described that inorganic sources tend to dissociate in the upper part of the gastrointestinal tract, causing a decrease in the availability of copper due to its interaction with other metals (chelation) and therefore a reduction in its activity [18, 45]. In contrast, the solubility of organic sources of copper is higher in weak acid enviroments, making their dissolution slower and increasing their availability and activity [45]. Furthermore, lower fecal copper excretion rates have been reported in broilers exposed to an organic source of copper compared to inorganic sources [46]. Although the copper ion is known to be more effective against Gram-positive bacteria [47], dietary supplementation with CA significantly reduced more than 18% the colonization of *S*. Typhimurium in both trials compared to PC group (Table 2). Copper ion has been reported to cause damage at the bacterial membrane level due to its adhesion to membranes and the generation of reactive oxygen species [48]. Additionally, it can be associated with the functional groups of proteins and enzymes, leading to the inactivation or inhibition of some cellular processes, as well as having a direct negative effect on the genetic material of bacteria [47, 48]. In addition, this reduction in the colonization of *S.* Typhimurium presented a positive effect on BW and BWG since they tend to improve in both experiments (*P* = 0.081 and *P* = 0.085, respectively), when compared to PC.

In the case of the group treated with the solid dispersion of curcumin (CR), which was previously described by our research group and is characterized by being more soluble and permeable [22], the colonization of *S*. Typhimurium significantly decreased by more than 35% (more than 2 log_10_) with respect to the PC group after 10 days of treatment (Table 2). These results are due to the antimicrobial action of curcumin, which in general, is associated with damage to the bacterial membrane and inhibition of bacterial cell proliferation [49, 50]. Furthermore, it has been published that curcumin can induce some physical and mechanical changes of the *S*. Typhimurium flagellar filament, causing a decrease in motility, adherence, and invasion of the host cells, which results in a reduction or elimination of its virulence [51]. Likewise, curcumin has been reported to decrease bacterial cell division processes since it interacts with the FtsZ protein, a cytoskeleton protein essential for this process [52]. The treatment containing the mixture of CA and CR (CA-CR) reduced 2% and 37% the *S.* Typhimurium colonization compared to the group treated with CR and the PC group, respectively. These results contrast with those obtained in other articles where the combination of curcumin with heavy metals, including copper showed better effects and even decrease the toxicity of metals [16, 53, 54].

After oral infection with *Salmonella*, this pathogen must overcome the conditions of the gastrointestinal tract to interact with the intestinal epithelium [55]. Invasion of epithelial layers by *S*. Typhimurium is known to increase intestinal permeability in both *in vivo* and *in vitro* models since the expression of some markers such as claudin-1, occludin, and mucin-2, mRNA levels of zonula occludens-1 and E-cadherin was reduced [55, 56]. In the present study, FITC-d, a large molecule (3–5 kDa) that, under normal intestinal health conditions, does not leak through the epithelium, was used to assess intestinal permeability. However, when there is damage to the epithelium, the permeability of FITC-d increases so that it can be quantified in serum [57]. In the present study, all treated groups showed lower serum FITC-d concentrations compared to the PC group (Table 2). However, only the group treated with CR had significantly lower concentrations when compared to PC and turned out to have serum FITC-d concentrations comparable to the NC group. Perhaps, this result is due to the ability of CR to restore the intestinal barrier function and the expression of proteins associated with the tight junctions, the proliferation-regeneration of the intestinal epithelium, and its antimicrobial action, resulting in decreased paracellular permeability as has been previously reported [58, 59]. Regarding the treatments with CA and CA-CR, although the *S.* Typhimurium counts decreased significantly compared to the PC group, the serum FITC-d concentration only decreased numerically since it has been described that the production of reactive oxygen species by copper affects not only bacteria but also epithelial cells [60].

The chicken gut microbiota are densely populated with complex microbial communities that are involved in digestion and metabolism, regulation of enterocytes, vitamin synthesis, and development and regulation of the host immune system [61]. Cecum is by far the most densely colonized microbial habitat in chickens [62]. Despite the absence of any clinical signs of *Salmonella* infection, it has been reported that the composition of the microbiota is affected, but the changes are quite weak at the level of the caecal tonsils [63, 64], which supports our results since no significant differences in alpha (measured by the observed OTUs) and beta diversity were observed in the cecal samples at day ten post-*S*. Typhimurium challenge. This means that there were no changes in the relationship of the number of different species per sample (richness) and in the diversity of the microbial community between different samples, respectively [65]. Notwithstanding the above, the taxonomic composition showed some significant differences at the family and genus levels when the groups were compared.

At the family level, abundance of Enterococcaceae was lower in all groups supplemented with CA when compared to the PC group. Enterococcaceae, one of the six families of the order Lactobacillales [66], is comprised of the genera *Enterococcus*, *Bavariicoccus*, *Catellicoccus*, *Melissococcus*, *Pilibacter*, *Tetragenococcus*, and *Vagococcus* [67]. However, it has been described that the dietary copper supplementation alters the intestinal microbiota, decreasing the abundance of Enterococcaceae due to the total reduction of lactic acid bacteria [68]. In contrast, *Salmonella* infection is known to increase the relative abundance of Enterococcaceae, Lactobacillaceae, Clostridiaceae, Lachnospiraceae, Erysipelotrichaceae, Peptostreptococcaceae, and Ruminococcaceae, but decrease that of Enterobacteriaceae [69]. Furthermore, the family of Clostridiaceae was significantly lower in chickens whose diet contained CR in common compared to PC. Clostridiaceae is one of the responsible families for converting polysaccharides into short-chain fatty acids (SCFAs) [70]. It has been described that SCFAs such as acetate, propionate, and butyrate, are important in maintaining intestinal homeostasis due to their immunomodulatory capacity, maintenance of metabolism, proliferation, differentiation and promotion at low pH, favoring beneficial bacteria, and reducing the growth and viability of pathogenic bacteria [71]. Therefore, these results support the lower *Salmonella* counts in CT and the improvement in BW and BGW in the CR treated group.

At the genus level, *Salmonella*, *Coprobacillus*, *Eubacterium*, and *Clostridium* were significantly enriched in the PC group, which is closely related to the severity of the *Salmonella* infection process. *Coprobacillus*, *Clostridium*, and *Eubacterium* have an important role in the production of SCFAs essential amino acids and the digestion of non-starch polysaccharides, which stimulate the production of SCFAs for metabolic balance [70, 72]. Likewise, it has been reported that the reduction of *Clostridium* and the maintenance of *Eubacterium* and *Coprobacillus* levels could be related to the effectiveness of the treatments since they represent a positive effect in the maintenance of intestinal homeostasis [72–74]. Regarding the high abundance of *Salmonella*, it has been reported that it is related to its colonization in CT [75]. Although sequencing of the V4 region of 16S rRNA gene is not able to distinguish between Enterobacteriaceae, BLAST analysis using an amplicon sequence variant (ASV) that matched to genus *Salmonella* strongly supports that the taxonomic assignment of this ASV to genus *Salmonella* in this study was accurate (see Supplementary Materials). Hence, these results confirm again the effect of the treatments, especially CR, on the decrease in *Salmonella* counts, the maintenance of intestinal integrity as indirectly measured by the serum FITC-d concentration, and the improvement in BW and BWG.

Furthermore, the genus *Faecalibacterium* and *Enterococcus* were significantly enriched in the group treated with CR. After infection with *Salmonella*, this pathogenic bacteria alters the intestinal microbiota, causing a decrease in bacteria of the genus *Blautia*, *Enorma*, *Faecalibacterium*, *Shuttleworthia*, *Sellimonas*, *Intestinimonas*, and *Subdoligranulum*, as well as an increase in the abundance of *Butyricicoccus*, *Erysipelatoclostridium*, *Oscillibacter* and *Flavonifractor* [61]. However, in the case of the group treated with CR, the increase in *Faecalibacterium*, a genus of bacteria responsible for the production of butyrate and related to health benefits in poultry, could be mainly due to the prebiotic effect of curcumin, like other substances with the same activity [76]. It has been described that CR could act as a factor of promotion, proliferation, growth, and survival for the beneficial bacteria of the intestinal microbiota from its biotransformation [77]. Finally, the bacterial genera that belong to Erysipelotrichaceae and Lachnospiraceae were significantly enriched in the CA-CR and CA groups, respectively. It has been published that in chickens infected with *Salmonella* this genus of bacteria decreases markedly, which could negatively affect the diversity and development of intestinal bacteria [69]. In the specific case of CA and CA-CR, copper is known to increase the relative abundance of these bacterial genera, which are the most active microbial components in the healthy gut and are responsible for preventing the production of inflammatory cytokines and induce intestinal production of SCFAs by fermenting carbohydrates [78, 79]. Although the sample size for microbiome analysis is small, the results are promising and suggestive since there is a close relationship with what was observed in the other determinations.

## Conclusion

According to the previous results, it can be concluded that the treatment with CR was the most effective in reducing *S*. Typhimurium counts. Furthermore, it was determined that the antimicrobial activity of CR, when administered at 0.2% into the feed using an *S*. Typhimurium infection laboratory model, is based on a combined mechanism in which direct activity on pathogenic bacteria and the prebiotic effect is mainly involved. Finally, it is clear that physical mixtures of CR with a metal such as copper (CA) are not effective, increasing the antimicrobial effect. Studies to confirm and expand these results with a larger number of animals and samples, and considering the analysis of inflammatory and antioxidant biomarkers to get a complete description of CR required further investigation.

## Supplementary Information

**Additional file 1.** Identification of genus *Salmonella* in microbiota analysis

## Data Availability

The sequencing data of cecal microbiota is available on NCBI Sequence Read Archive (SRA) under BioProject number BioProject ID PRJNA655142.

## Abbreviations

BGA: Brilliant green agar
CT: Cecal tonsils
CA: Copper acetate
CR: Curcumin
CA-CR: Copper acetate and curcumin
FITC-d: Fluorescein isothiocyanate dextran
LEfSe: Linear discriminant analysis effect size
NA: Nalidixic acid
NO: Novobiocin
SCFAs: Short-chain fatty acids
TSB: Tryptic soy broth
NC: Negative control
PC: Positive control
XLT-4: Xylose Lysine Tergitol-4
