# Supplementary material for: Evaluation of curcumin and copper acetate against Salmonella Typhimurium infection, intestinal permeability, and cecal microbiota composition in broiler chickens
Source: J Anim Sci Biotechnol. 2021 Feb 5;12:23. doi: 10.1186/s40104-021-00545-7 (PMC7863265; doi:10.1186/s40104-021-00545-7)

**Supplementary Materials**

**Evaluation of curcumin and copper acetate against *Salmonella* Typhimurium infection, intestinal permeability, and cecal microbiota composition in broiler chickens**

Anaisa A. Leyva-Diaz^1^, Daniel Hernandez-Patlan^2^, Bruno Solis-Cruz^2^, Bishnu Adhikari^3^, Young Min Kwon^3^, Juan D. Latorre^3^, Xochitl Hernandez-Velasco^1^, Benjamin Fuente-Martinez^4^, Billy M. Hargis^3^, Raquel Lopez-Arellano^2^ and Guillermo Tellez-Isaias^3^*

**Identification of genus *Salmonella* in microbiota analysis**

In this study, we orally gavaged all day-of-hatch chicks in all groups except for NC with *S.* Typhimurium PHL-2020 strain. When we analyzed MiSeq sequence reads of 16S rRNA genes from cecal samples using DADA2 algorithm in QIIME2, we detected only one amplicon sequence variant (ASV) that matched to genus *Salmonella*. The sequence of this ASV (373 nucleotides long) is shown below:

TACGGAGGGTGCAAGCGTTAATCGGAATTACTGGGCGTAAAGCGCACGCAGGCGGTCTGTCAAGTCGGATGTGAAATCCCCGGGCTCAACCTGGGAACTGCATTCGAAACTGGCAGGCTTGAGTCTTGTAGAGGGGGGTAGAATTCCAGGTGTAGCGGTGAAATGCGTAGAGATCTGGAGGAATACCGGTGGCGAAGGCGGCCCCCTGGACAAAGACTGACGCTCAGGTGCGAAAGCGTGGGGAGCAAACAGGATTAGATACCCTGGTAGTCCACGCCGTAAACGATGTCTACTTGGAGGTTGTGCCCTTGAGGCGTGGCTTCCGGAGCTAACGCGTTAAGTAGACCGCCTGGGGAGTACGGCCGCAAGGTTA

The frequency of this AVS reads was total 695 in 7 samples as shown in the table below.

| Index | Treatment | Before rarefaction | | After rarefaction | |
| --- | --- | --- | --- | --- | --- |
|  |  | genus Salmonella | Total read counts | genus Salmonella | Total read counts |
| ANA-1-1 | NC | 0 | 94583 | 0 | 69566 |
| ANA-1-2 | NC | 0 | 75139 | 0 | 69566 |
| ANA-1-3 | NC | 0 | 94729 | 0 | 69566 |
| ANA-1-4 | NC | 0 | 121233 | 0 | 69566 |
| ANA-1-5 | NC | 0 | 109204 | 0 | 69566 |
| ANA-2-1 | PC | 47 | 83388 | 43 | 69566 |
| ANA-2-2 | PC | 63 | 100502 | 41 | 69566 |
| ANA-2-3 | PC | 165 | 96924 | 123 | 69566 |
| ANA-2-4 | PC | 159 | 102306 | 110 | 69566 |
| ANA-2-5 | PC | 236 | 111518 | 138 | 69566 |
| ANA-3-1 | CA | 0 | 104855 | 0 | 69566 |
| ANA-3-2 | CA | 0 | 78143 | 0 | 69566 |
| ANA-3-3 | CA | 0 | 69566 | 0 | 69566 |
| ANA-3-4 | CA | 0 | 113760 | 0 | 69566 |
| ANA-3-5 | CA | 0 | 81440 | 0 | 69566 |
| ANA-4-1 | CR | 0 | 118198 | 0 | 69566 |
| ANA-4-2 | CR | 0 | 144899 | 0 | 69566 |
| ANA-4-3 | CR | 0 | 112376 | 0 | 69566 |
| ANA-4-4 | CR | 0 | 132693 | 0 | 69566 |
| ANA-4-5 | CR | 0 | 126829 | 0 | 69566 |
| ANA-5-1 | CA-CR | 0 | 150582 | 0 | 69566 |
| ANA-5-2 | CA-CR | 25 | 114467 | 14 | 69566 |
| ANA-5-3 | CA-CR | 0 | 155679 | 0 | 69566 |
| ANA-5-4 | CA-CR | 0 | 148901 | 0 | 69566 |
| ANA-5-5 | CA-CR | 0 | 127595 | 0 | 69566 |
| Total |  | 695 |  | 469 |  |

To assess the accuracy of the taxonomic assignment for this ASV, we performed BLAST analysis of this ASV sequence against the major genera in family Enterobacteriaceae, *Escherichia*, *Salmonella* and *Shigella* in Nucleotide collection (nr/nt) using default settings. The BLAST results below showed that this ASV has 100% identity (373/373) with genus *Salmonella*, while it was lower with genus *Escherichia* (98.93-99.20%) and *Shigella* (97.32-99.20%). The result strongly supports that the taxonomic assignment of this ASV to genus *Salmonella* in this study was accurate.

Top 10 hits in genus *Escherichia*


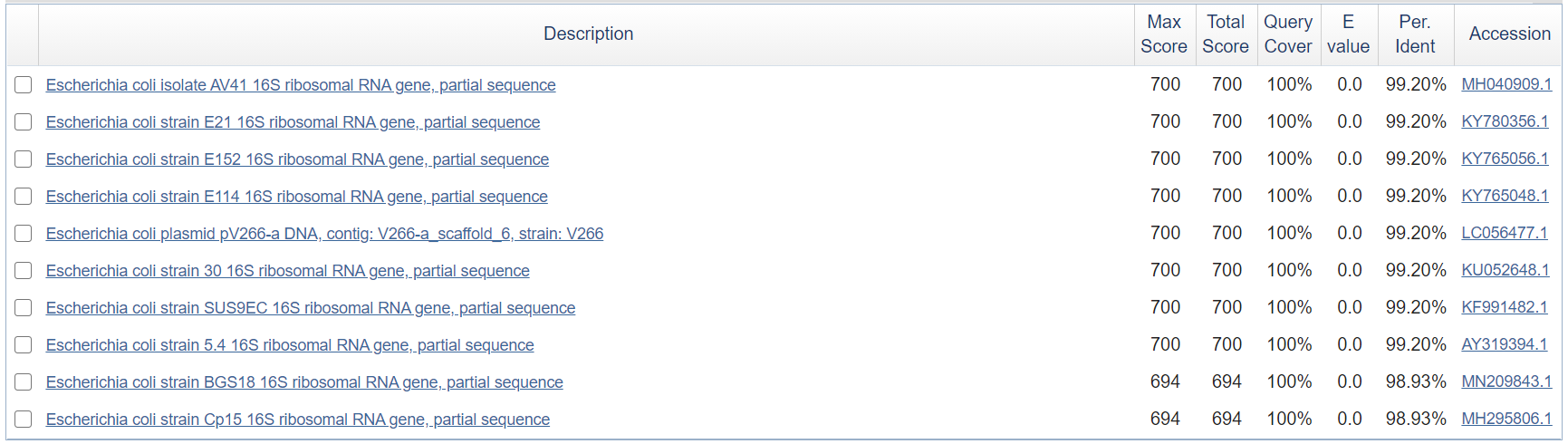


Top 10 hits in genus *Salmonella*


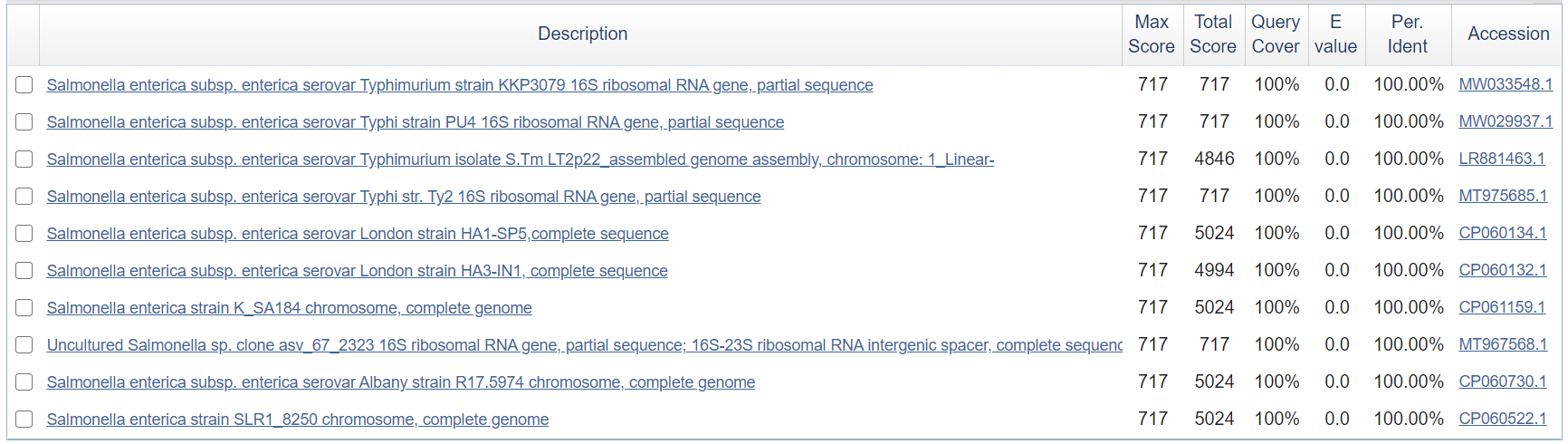


Top 10 hits in genus *Shigella*


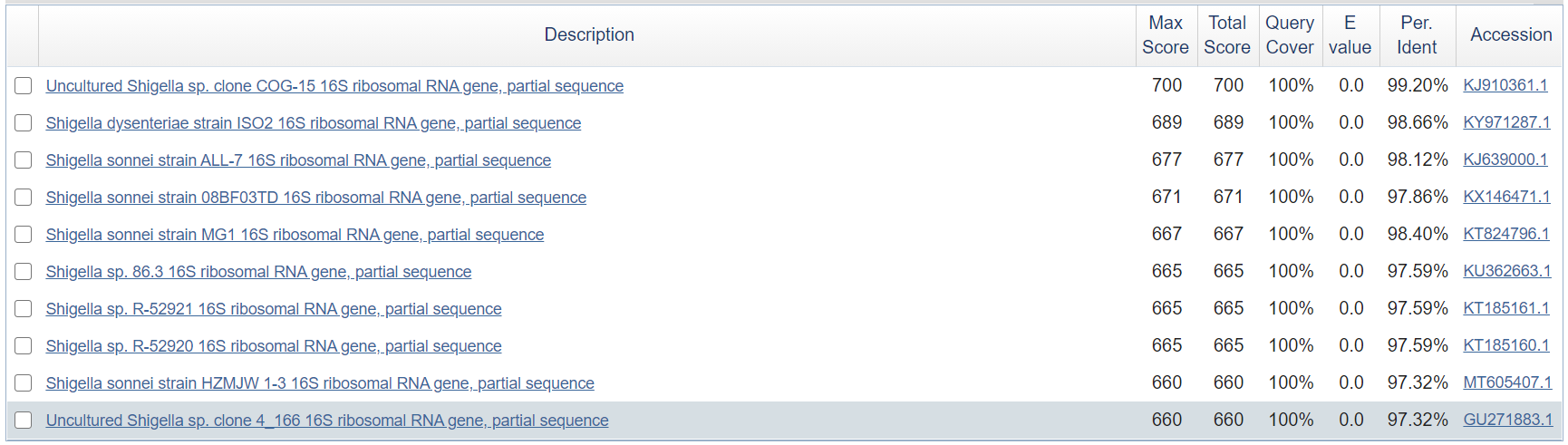

Supplement: Supplementary file 1 — Additional file 1. Identification of genus Salmonella in microbiota analysis [file 40104_2021_545_MOESM1_ESM.docx]
